# Near miss road traffic accidents and associated factors among truck drivers in Gamo zone, southern Ethiopia by using a contributory factors interaction model

**DOI:** 10.3389/fpubh.2024.1386521

**Published:** 2024-07-24

**Authors:** Tariku Bekelcho, Gebremaryam Temesgen Birgoda, Hawi Leul, Maechel Maile, Micheal Alemayehu, Ararso Baru Olani

**Affiliations:** ^1^Department of Emergency Medicine and Critical Care, College of Medicine and Health Sciences, Arba Minch University, Arba Minch, Ethiopia; ^2^Department of Midwifery, College of Medicine and Health Sciences, Arba Minch University, Arba Minch, Ethiopia; ^3^Department of Emergency and Critical Care, Tirunesh Beijing General Hospital, Addis Ababa, Ethiopia

**Keywords:** near-miss accident, road traffic accident, truck drivers, Gamo zone, banana transportation

## Abstract

**Background:**

Road traffic accidents (RTAs) are among the leading causes of injuries, fatalities, and the resulting increase in financial burdens worldwide. Every year, RTAs cause numerous serious injuries and fatalities in Ethiopia. it is important to understand how prevalent near-miss crash accidents are, and which by definition could have injured the victim but did not result in an actual accident. The determinants of these near-misses are essential in road crash accident reduction strategies. In spite of the fact that near-miss accidents are much more common than actual losses or injuries, very little research has been conducted on them. Thus, this study was intended to assess the near-miss accidents and associated factors among truckers in Gamo zone, southern Ethiopia.

**Methodology:**

The community-based cross-sectional study was employed from May 12 to July 10,2022, using a structured interviewer-administered questionnaire. A simple random sampling technique was used to select participants. The data were analyzed using the statistical package for social sciences. A binary and multivariate logistic regression model was used to identify the determinants of near-miss accidents. A statistical significance level was set at *p* < 0.05.

**Results:**

About 72.5% of truckers had experienced near-miss road traffic accidents. The majority of the near-miss accidents were caused by speeding, followed by driving on the wrong side of the road and skidding, 65 (22.6%), 39 (13.5%), and 38 (13.2%), respectively. Driving frequency per week, location of accidents, condition of the road, sleeping status, and weather conditions were significantly associated with near-miss accidents.

**Conclusion:**

The prevalence of near-miss accidents is high in the Gamo zone. Being a younger and less educated driver, high driving frequency per week, driving on major roads and junctions, foggy weather, and inadequate sleep all contribute to the occurrence of accidents. Road safety measures that could address these identified factors are required to mitigate potential RTAs.

## Introduction

The number of deaths from road traffic accidents (RTAs) is still higher globally, with an estimated 1.35 million people dying each year and over 3,500 road deaths occurring daily (1). This makes RTAs the eighth leading cause of death for all age groups and the first leading cause of death for children and young adults aged 5 to 29 years old from neglecting RTAs from a child health agenda. Undesirably, the risk of RTA death is three times higher in low-income countries than in high-income countries (1, 2).

Also, these accidents could often result in injuries, disabilities, and a financial burden that could affect victims and society in general (3, 4). In addition, global RTAs are estimated to cost countries $1.8 trillion in 2015–30, while economic losses in low- and middle-income countries are estimated at $1 trillion a year. Despite the fact that RTA is both predictable and avoidable, it remains a daily event in many countries around the world. More regrettably, the efforts and investments made so far have not attained the desired goal of reversing the tragic impacts of the RTA and the target of halving the number of global deaths and injuries from the RTA by 2020 (3, 5, 6).

In Africa, RTA deaths are highest, representing 26.6/100,000 people, due to the poor enactment and enforcement of key risk factors including speeding, drunk driving, motorcycle helmet use, use of seatbelts, and child restraints (1, 2). Similarly, in Ethiopia, the mortality rate associated with RTAs was higher, representing 37/100,000 persons in 2018 (7). RTAs remain excessively high, and they most frequently affect approximately half (47.1%) of the young adults in the 15 to 29 years old age group in Ethiopia (8, 9).

Numerous studies that have been conducted on RTAs have identified the factors related to human, vehicular, and environmental factors attributed to actual RTAs (7–11). Likewise, studies conducted on RTAs in Gamo zone reported that RTAs were mainly caused by human errors such as overloading, speeding, fatigue driving, and drunk driving. However, these studies were lacking in the consideration of an assessment of the road safety system in general including outside factor. Instead, these studies were focused primarily on human errors (8, 9). Furthermore, the impacts of near-miss accidents on the occurrence of actual crash accidents were not incorporated into these studies.

The notion that individual road users are solely responsible for the occurrence of RTAs stems from the findings of the World Health Organization in 2004, which assert that human error is the main cause of about 95% of RTAs (12). Consequently, corrective actions were initially sought to persuade road users to adopt error-free behaviors. These measures consisted of information and education in order to ensure that road users behave correctly and regulate and monitor their behaviors (13). In contrast, the occurrence of crash accidents is not solely attributed to human behavior. Thus, this suggestion excluded other determinants of crash accidents.

It has been reported that accidents occur when components of a system interact with each other, and these interactions are hard to predict because of their complexity. Thus, safety can be improved by understanding the nature of an accident rather than focusing on its causes (14). Moreover, safety cannot be optimized by improving the safety performance of individual components. It is often unsuccessful to improve safety in complex systems by examining and modifying individual components (15). As a result, some authors assert that recognizing accidents and determining the appropriate remedial actions require a comprehensive examination of the entire system as opposed to focusing on individual components (16).

Based on these suggestions, the current study attempted to assess the nature of accident environments and conditions, in addition to road users’ behaviors. These might help to figure out how accidents occur and how safety systems work.

Furthermore, the chance of RTA increases with near-miss crash incidents. For drivers who experienced four or more near-miss accidents (NMAs), the probability of sustaining RTAs increased by twofold compared to their counterparts (4, 17).

Near-miss road traffic accidents are events with potential safety-related effects that, at the end, were prevented from evolving into actual crash accidents (18). That is the reason the drivers usually consider it almost an accident and produce more than an ordinary amount of danger to them or their passengers. Consequently, it is memorable as an accident to the driver that he or she can still recall the details of the situation (6, 19).

Previous studies showed that, among half of the respondents who had experienced near-miss accidents within the preceding year, more than two-fifths of them had sustained at least one actual RTA (4, 17, 19).

Scholars have investigated the correlation between NMAs and driving experience, poor attention performance, sleep quality, vehicle conditions, and drivers’ health status (17, 20–23).

Scholars have reported a high prevalence of RTAs and identified associated factors in Ethiopia (7, 8, 24). However, no study has been conducted to investigate pre-accident conditions (near-miss accidents) and their determinants by incorporating external factors using a safety model as a framework in the study area.

Thus, the current study was conducted by incorporating some additional variables in order to bridge the gap. These variables include a history of speeding tickets, periodic training opportunities, enforcement of rules and regulations on road safety, and a reporting system at the time of health difficulties while driving. Furthermore, the contributory factor interactions model (CFIM) was used, and its components were evaluated. The assessed parameters comprise outside factors, organizational influence factors, unsafe supervision, and a precondition for unsafe behaviors.

Improving the components of the safety system and studying the nature of accidents are more successful in accident prevention than focusing only on the causes of accidents (14). Identifying risk factors for NMAs in various components of the road safety system is essential for establishing road traffic injury prevention strategies (17, 20, 21).

Thus, this study aimed to accomplish two objectives. (i) To determine the magnitude of near-miss accidents. (ii) To identify the determinants of the near-miss accidents among truckers. The prevalence of NMAs and a statistically significant association between the response variable (near-miss accident) and explanatory variables were computed using statistical software and various modeling techniques.

**Figure 1:**
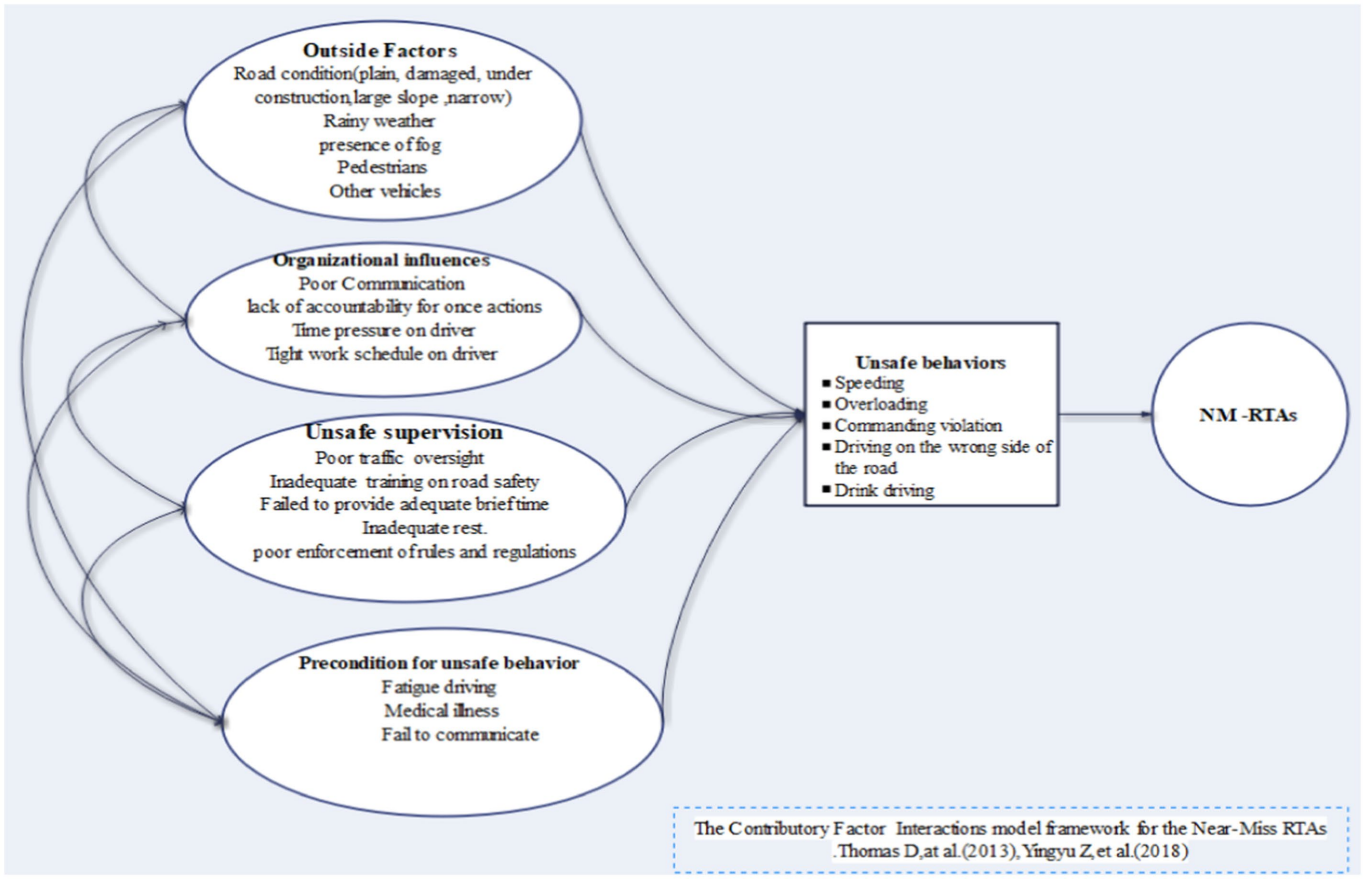
Contributory factor interactions model framework for the near-miss accidents among truckers in Gamo zone, southern Ethiopia (21, 25).

In this research, using the Statistical Package for the Social Sciences (SPSS) software, the logistic regression modeling method was used to investigate the relationship between outcome variable and independent variables. A diagram that illustrates the detailed lists of selected explanatory variables from CFIM is presented in Figure 1.

The methods used and steps followed were presented in Section III. This includes determining the size of study participants using a single population proportion formula, setting inclusion and exclusion criteria, and describing the level of statistical significance of the explanatory variables. Section IV discusses the details of the results of this study. The demographics of the subjects, near-miss and crash accident-related data, and factors associated with the occurrence of NMAs are all described in this section. Section V provides a thorough discussion, and Section VI concludes the research work with a recommendation.

### Overview of related works

It has been reported that perceived traffic risks are multi-faceted. Also, it was demonstrated that knowing the dynamics of near-miss accidents while driving could be critical for understanding the perceived traffic risks. Moreover, it has been indicated that the frequency of driving and driving on the busiest street increases the probability of experiencing near-miss accidents, which occur much more often than actual crashes. Furthermore, it was suggested that attempts to understand road traffic risk for drivers through reported crash data alone may underestimate the risks of road traffic accidents (26).

The relationship between time spent working as a trucker and involvement in RTA or near-miss accidents was investigated. It was found that longer professional experience and a reduction in reporting involvement in crash accidents and near-miss accidents. In the same study, working conditions, lifestyle, health conditions, use of psychoactive substances, accident involvement, and other factors affecting truck drivers have been studied so far. According to the findings of this study, 48 drivers (7.2%) experienced traffic accidents, and 41.7% of drivers were involved in near-miss accidents during the last 12 months. This is a clear indication of how common NMAs are among truckers compared to actual crash accidents, which need proper interventions. But this study did not include information about the condition of the roads and vehicles at the time of the accident. The possible suggested strategy to reduce crashes or NMAs among truckers would be improving trucker training (17).

A study done in Japan among older drivers evaluated an association between experience of near-miss incidents, attention, and executive function. It was found that the prevalence of near-miss accidents was significantly higher among men than women. Also, male gender, high driving frequency, and poor attention were associated with near-miss incidents. This study used neuropsychological tests to assess attention and processing speed, as well as sensorimotor function. However, it has limitations in assessing sensorimotor function using reaction and movement times to examine the effects of sensorimotor function on near-miss incidents (23).

A cross-sectional study done in Nigeria among commercial tricycle drivers revealed that drivers who had experienced near-miss accidents occurred in unfavorable weather, on the road, and while feeling sleepy. This study found that having experienced more than one NMA significantly predicted having crash accidents (27).

A cross-sectional study conducted in Thailand among interprovincial public van drivers reported that the major causes of near misses were swerve or brake, loss of grip, and skidding. This study discovered that more working experience, extended work hours, reckless driving behavior, smoking, and poor sleep quality were the risk factors for near-miss accidents (21).

Another study, conducted in Japan, among taxi drivers, evaluated the impact of workplace environment, working conditions, and drivers’ health status on vehicle collisions or near-miss events. In this study, event groups were those who had experienced collisions or near-miss incidents caused by health problems, and non-event groups were those who had no accidents. It was found that being unable to take vacation time, having chronic diseases, taking insufficient vacation time, and difficulty reporting poor health conditions were significantly associated with the occurrence of accidents (20).

Moreover, a previous study illustrated that the near-miss incidents were higher among participants with higher fatigue. According to the findings of this study, fatigue was significantly associated with frequent ambulance dispatches, long office working hours, irregular mealtimes, and irregular sleep patterns at different working shifts. These lifestyle irregularities are a root cause of fatigue in ambulance personnel (28).

The authors also examined the factors involved in drowsy driving that can cause collisions or near-miss events among bus drivers in Ibaraki Prefecture. The result of this study revealed that driving when feeling sick was a major risk factor for collisions and near-miss incidents among drivers. The recommendation to divert this problem was to create a good working environment that encourages drivers to voluntarily and smoothly report any health problems they may have (22).

More importantly, numerous studies have been conducted on different risk factors related to crash accidents and near-misses. Several suggestions have been made to address these issues. An embedded in-vehicle automatic accident and barrier detection notification system was developed as part of the solution. Two novel algorithms were presented, namely, a radar range algorithm for barrier detection on the road before collision occurrence. And a distance-time-based parameter algorithm for detecting accident geolocation coordinates. These algorithms are instrumental in detecting crash accidents and roadside barriers that may potentially lead to accidents by providing an earlier warning prior to the occurrence of a crash. The developed system was tested, and it was found to be effective in reducing the mortality rate among victims of crash accidents (29).

The vehicle tracking system for public transportation on urban arterials was developed, implemented, and demonstrated using innovative algorithms. This system would help prevent vehicles from being diverted to unauthorized routes and enhance fleet management. Such a system that enhances fleet management would have a crucial role in improving visibility of geographic locations and monitoring vehicle speed. The control of vehicle speed would play an essential role in reducing the incidence of road crash accidents and improving the road safety system (30) (Table 1).

However, studies conducted so far have lacked assessments of outside factors, organizational influence factors, unsafe supervision, a precondition for unsafe behaviors, and unsafe behaviors. Identification of these factors as a whole system would enhance efforts to find appropriate preventive measures at the pre-accident level before an actual accident happens. Thus, the current study assessed these factors to identify determinants of near-miss accidents among truckers.

## Materials and methods

### Study setting

This study was conducted in the Gamo zone, which is located in the Southern Nation, Nationalities, and People’s Region of Ethiopia. Gamo zone is located in southwest Ethiopia and is one of the best tourist destinations. The total population of the zone is 1,613,513 (female, 810, 956 = 50.26% and male, 802,557 = 49.74%). Arba Minch town is the capital city of the Gamo zone, located 505 km away from Addis Ababa and 275 km southwest of Hawassa. The number of registered vehicles in the zone is 4,431, with motorcycles (66.6%), tricycles (3.4%), passenger cars (19.5%), taxis (6.9%), and other vehicles (2.84%) in the zone (24). The Gamo zone has a tropical climate with a bimodal rainfall pattern, with a minor rainy season (September–November) and a major rainy season (March through May). After having a rainy season from March to May, this area becomes very hot, and bananas produce more fruits in the hot weather. The annual average temperature ranges from 15°C to 28°C. Moreover, there is a similar pattern of banana harvest in the area without significant variation in the seasons (31, 32). Banana is an important fruit crop in the Gamo zone, and over 65% of bananas commercially sold in all major towns in Ethiopia are produced in this zone. In the Gamo zone, the area allocated for banana production is significantly higher than other banana-growing zones. The irrigated production system is the predominant method of production among all producers in Gamo zone, whereas all farmers in other regions of the country follow the rain-fed system. It is a major component of livelihood strategies and plays a considerable role in household nutrition and income generation in the area (33). Depending on the season, 60 to 90 truckloads of banana bunches are dispatched every day, each truck carrying 5–9 tons (Isuzu trucks 5–5.5 tons and Isuzu FSR trucks 8–9 tons), and transported on piled-lose bunches from this zone to all market outlets in Ethiopia (33, 34).

### Study design and period

This community-based cross-sectional study was employed from May 12 to July 10, 2022.

### Study participants and sampling procedure

The study participants were all truckers involved in the transportation of the bananas from harvest in the Gamo zone who agreed to participate in the study. We selected this group of study participants purposefully, for some reason. Truckers drive heavy trucks for longer hours without breaks, with loads of 5–9 tons per trip. Also, their working time is usually nighttime shifts from banana farmland to marketplaces, literally from 6:00 pm to 5:30 a.m., which is different from the working shifts of other groups of drivers. The aforementioned factors are anticipated to augment the likelihood of engaging in risky driving behaviors, as there is no presence of traffic police inspection and supervision during the night. Considering the preceding factors, it is posited that this particular group of drivers is assumed to be more susceptible to sustaining near-miss crash accidents than other groups of drivers and has been selected to be investigated.

The sample size was determined by using the single population proportion formula, which incorporated the following assumptions: 38.3 prevalence of near-miss road traffic accidents from the previous study (21), a 5% margin of error, and a 95% confidence interval. This calculation gave a sample size of 363, which was then increased by 10% to account for non-responses. As a result, the final sample size was 399.

The selection of study participants involved identifying the banana transportation truck drivers in Gamo zone. To select the drivers, we obtained a list of drivers and their trucks from the truck drivers and truck owners associations in the Gamo zone, Arba Minch town. This list of truck drivers by their plate number and driver’s phone was used to create a sampling frame, which is a list of all truck drivers involved in banana transportation that could potentially be included in the sample. Ultimately, 399 drivers were selected through simple random sampling, using a table of random numbers generated in SPSS using the drivers’ identification numbers. Selected participants were reached through their phone numbers and the dispatch center of their association. Trained data collectors with health backgrounds (nurses and public health officers) collected the data. In the event that participants were absent, drivers were visited three times to reduce non-participation.

### Data collection tool and measurements

Data were collected using a pretested and structured questionnaire that was adopted from previous literature and modified to fit with the study objectives (20, 35). It has included questions regarding sociodemographic characteristics and accident-related factors. To check for the reliability of the modified questionnaire, an inter-item reliability analysis was done using SPSS. The result of the internal consistency analysis (Cronbach’s alpha) of our modified questionnaire was 0.837, which was a good score that showed that the reliability of our tool is acceptable. The validity of our tool was maintained by having a high reliability score, extensive training for data collectors and supervisors, and close supervision at the time of data collection. The majority of the questions on the driver’s behavior and accident-related factors are taken from previously validated tools (17, 20, 35).

There is a scale of danger that could be applied to the traffic events to facilitate objective measurement and detection of NMAs. The danger scale utilized to measure NMAs objectively is the time measured until a collision between two vehicles involved in the unsafe event. This measure, computed from films taken with the traffic sensing and surveillance system, is an adequate unit to rate the danger of almost any traffic event, including near-miss accidents (6).

In our case, we have no such established traffic sensing and surveillance system around our main roads. So, getting the recorded films of the traffic movements to determine the prevalence of the NMAs in our study area was not feasible. Additionally, it could be challenging to get NMA related-recorded data from police officers instead of truckers because our study participants drive trucks between banana farms and marketplaces at night. Consequently, the NMAs related data was assessed from drivers who had experienced such incidents within the last 1 year and identified the underlying factors that contributed to the accidents. They were able to retain these events with a high degree of recall.

Moreover, different techniques were used to reduce a recall bias and social desirability bias among participants. The methods used include: questions were defined and phrased carefully, Interviewers were well-trained on data collection methods and data collection tools, and a conducive environment was created for study participants at the time of data collection. Also, a questionnaire was translated into the local language and pre-tested to clear up all misunderstandings and difficulties for both participants and interviewers. All interviewees were allowed sufficient time for adequate recall of accident-related memories from the last year at the time of data collection (36, 37).

In the investigation and analysis of accidents, an accident causation model plays a pivotal role (20). Using safety models is advantageous in establishing rules, checking, evaluating, determining, and assessing causation and communication (35). The CFIM was implemented to assess factors associated with near-miss road traffic accidents. The CFIM is a suitable conceptual model to assess the complex system of contributory factors identified in the accident reports. It is helpful to analyze and explain the linkages and relationships within and across the road transportation industry to identify common factors that interact across organizational and external factors (25).

According to CFIM, there are a number of determinant factors for the RTAs. These main factors assessed were: outside factors (damaged and narrow road, large slope, rainy weather, pedestrians and other vehicles), organizational influence factors (tight work schedule on driver), unsafe supervision (poor traffic oversight, failure to enforce rules and regulations, poor training on road safety), a precondition for unsafe behaviors (fatigue driving, medical illness, failure to communicate), unsafe behaviors (speeding, overloading, driving on the wrong side, drunk driving, command, and parking violation) (25, 38) (Figure 1).

It has been recognized that the investigation of the NMA is an important element in reducing the probability of the occurrence of major road traffic injuries by using accident causation models (6, 17, 21, 39). Thus, this study employed CFIM to assess factors associated with NMAs among truckers involved in the transportation of bananas in Gamo zone, southern Ethiopia.

### Data entry, processing, and analysis

The quality of the data was assured through careful design, training of the interviewers and supervisors, close supervision of the data collection procedures, and proper categorization and coding of the data. The data were entered into epi data version 7.2 and exported into SPSS for data analysis. Descriptive statistics, binary, and multivariate logistic regression analyses were employed to assess factors associated with NMAs. Statistical significance was declared for the variables with a *p*-value of less than 0.05. The data were summarized and presented in the form of proportions, frequency tables, and graphs.

**Table 1:** Related works analysis.

Title	Methods used	Strengths	Limitations	Application area
Perceived traffic risk for cyclists: The impact of near miss and collision experiences (26)	Online survey, focus groups discussion, and pilot testing were used	Demonstrated that the frequency of driving bicycles is significantly associated with experiencing a near miss. Indicated that attempts to understand traffic Risk through reported crash data alone may seriously underestimate the risks for crash accidents.	Lacked a specific time limit to preclude near-miss and collision experiences, which could introduce bias. Has the limitation of evaluating predictor variables related to the situations under which the accidents occurred.	Road safety
Professional experience and traffic accidents/near-miss accidents among truck drivers (17)	Cross-sectional study design was employed.	It was described that having a longer driving experience was inversely associated with involvement in crashes and near-miss accidents.	Sample selection was accomplished through convenience sampling, which limited the external validity of this study.	Road safety
Associations of Near-Miss Traffic Incidents with Attention and Executive Function among Older Japanese Drivers (23)	Cross-sectional study design was used.	Revealed that poor performance in attention has a significant association with near-miss accidents among older drivers.	A self-report questionnaire was used to collect data from participants with poor cognitive function that have possibility of recall bias about near-miss incidents.	Road safety and mental health research
Road traffic accidents, near-misses and their associated factors among commercial tricycle drivers in a Nigerian city (27)	Cross-sectional study	Assessed the vehicular character and drivers’ behavior in detail.	Visual problem data were collected via self-reporting instead of a visual acuity test.	Road safety research
Risk Factors of Near-Miss Road Traffic Incidents among Inter-Provincial Public van Drivers in Thailand (21)	Cross-sectional study design was employed	Sleep quality and perceived vehicle conditions were evaluated	Assessed health conditions were limited to drinking alcohol, smoking cigarettes, and sleep quality, regardless of considering other mental and medical conditions.	Road safety research
Influence of workplace environment, working conditions and health status of taxi drivers on vehicle collisions or near-miss events (20)	Cross-sectional survey was conducted	It was clearly presented that a significant number of drivers experienced near-miss accidents due to health-related problems.	Lacked cross-checking self-assessed health-related data with more reliable data sources, such as medical records. Inadequate data on the working environment influences.	Road safety and health care research
Near-miss incidents owing to fatigue and irregular lifestyles in Ambulance personnel (28)	Cross-sectional study design was used.	The relationship between fatigue and near-miss accidents was evaluated and reported.	Fatigue was evaluated by single instead of using other validated methods such as visual analog scales.	Road safety and lifestyle research
Risk Factors for Collisions and Near-Miss Incidents Caused by Drowsy Bus Drivers (22)	Questionnaire survey using paper forms.	It was indicated that continuing to drive while feeling sick was a major risk factor for near-misses.	Drowsiness was evaluated subjectively. Drivers were surveyed in a non-anonymous manner.	Road safety and health care research

### Operational definitions

Near-miss accident: An event that had potentially significant effects related to a road traffic accident, but prevented it from becoming a road traffic accident. It is an event which did not end up in a crash.

Road traffic accident: An accident happened when the moving vehicle collides or smashed with another moving or stationary vehicle, objects, animals or human being or rollover itself and drivers are survivor of an accident.

A trucker or truck driver: is someone who drives a truck to transport a banana from harvest to the marketplaces, and who is the survivor of an accident.

Banana transporting trucks: refers to the heavy commercial trucks (Isuzu F-series trucks) that are commonly employed for transporting bananas from the harvest to major cities and have a carrying capacity of 5–9 tons.

## Results

### Socio-demographic characteristics

A total of 399 study participants were selected and enrolled in the study, but because two of the study participants’ data were incomplete, we excluded them at the time of data entry and analysis. Thus, the study was conducted among 397 truck drivers, and the response rate was 99.5%. Above three-fourths of participants, 322 (81.1%) were in the age groups of 26 and 45. About 238 (59.9%) of drivers had secondary school education, and the majority of 268 (67.5%) participants were married, followed by single 104(26.2%) marital status. Nearly two-thirds 286 (72%) of the participants were employees (Table 2).

**Table 2:** Socio-demographic characteristics of the truckers in Gamo zone, southern Ethiopia, 2022 (*n* = 397).

Variables	Classifications	Frequency	Percentage (%)
Age	≤ 25 years old	47	11.8
	26–45 years old	322	81.1
	> 45 years old	28	7.1
Sex	Male	397	100.0
Religion	Protestant	121	30.5
	Orthodox	225	56.7
	Muslim	48	12.1
	Others	3	0.8
Educational level	Primary school	98	24.7
	Secondary school	238	59.9
	College and above	61	15.4
Marital status	Married	268	67.5
	Single	104	26.2
	Divorced	19	4.8
	Widowed	6	1.5
Monthly income	<1,000 ETB	15	3.8
	1,000–3,000 ETB	68	17.1
	3,001–4,000 ETB	47	11.8
	≥ 4,001 ETB	267	67.3
Drivers’ relationship with the truck	Owner	46	11.6
	Employee	286	72.0
	Relative’s truck	44	11.1
	Family’s truck	21	5.3

Others, Adventist, Jehovah witness, ETB, Ethiopian birr.

### Road traffic related factors

More than half (52.4%) of the drivers had experienced RTAs. About 37% of the trucks were gaining speed at the time of the accident. 27.9% of the drivers did not use seatbelts, and 13% of the drivers were using mobile phones at the time of accidents. Also, around half (51%) of accidents occurred in inadequate lighting conditions. About 45.2% of the drivers were fatigued and driving at the time of the accident. Moreover, half (50.4%) of the drivers had driven for 6 and above hours without taking breaks or rest (Table 3).

**Table 3:** Road traffic accident-related factors among truckers in Gamo zone, southern Ethiopia, 2022 (*n* = 397).

Variables	Categories	Frequency (%)	Percentage (%)
Ever experienced road traffic accident?	Yes	208	52.4
	No	189	47.6
Truck stuck with___?	Another vehicle	92	44.2
	Pedestrians	34	16.3
	Overturned	45	21.6
	Animals	31	14.9
	Others (tree, house, truck problem, skidding, horse cart, etc.)	6	2.9
Truck speed at the time of an accident?	Less than 60 Km/h. in urban areas	40	19.2
	60 km/h. and above in urban areas	49	23.6
	Less than 70 km/h. in rural areas	15	7.2
	70 km/h. and above in rural areas	104	50.0
Loss of consciousness	Yes	74	35.6
	No	134	64.4
Was the truck traveling at accident time?	Slowing	57	27.4
	Gaining speed	77	37.0
	Steady speed	72	34.6
	Stopped	2	1.0
Did the truck stopped?	Yes	124	59.6
	No	84	40.4
Seatbelt used	Yes	150	72.1
	No	58	27.9
Mobile phone used	Yes	27	13.0
	No	181	87.0
Was fog present?	Yes	72	34.6
	No	136	65.4
Was it raining when the accident happened?	Yes	49	23.6
	No	159	76.4
Lighting condition	Adequate	102	49.0
	Inadequate	106	51.0
Alcohol taken	Yes	19	9.1
	No	189	90.9
Stressed or fatigued while driving	Yes	94	45.2
	No	114	54.8
Animals on the road	Yes	117	56.2
	No	91	43.8
Runover by the truck	Yes	137	65.9
	No	71	34.1
Skidding happened?	Yes	72	34.6
	No	136	65.4
Any one died	Yes	89	42.8
	No	119	57.2
Have you been taking breaks during work?	Yes	392	98.7
	No	5	1.3
How many hours you have been working without breaks?	Less than 6 h	143	36.0
	6 h and above	200	50.4
	Unknown	54	13.6
Ever experienced accident-related injury?	Yes	137	34.5
	No	260	65.5
what was the nature of the injury?	Blunt	34	24.8
	Penetrating	12	8.8
	Cut or open	11	8.0
	Multiple injury	20	14.6
	Fracture	49	35.8
	Sprain	11	8.0

Km/h, Kilometer per hour.

**Figure 2:**
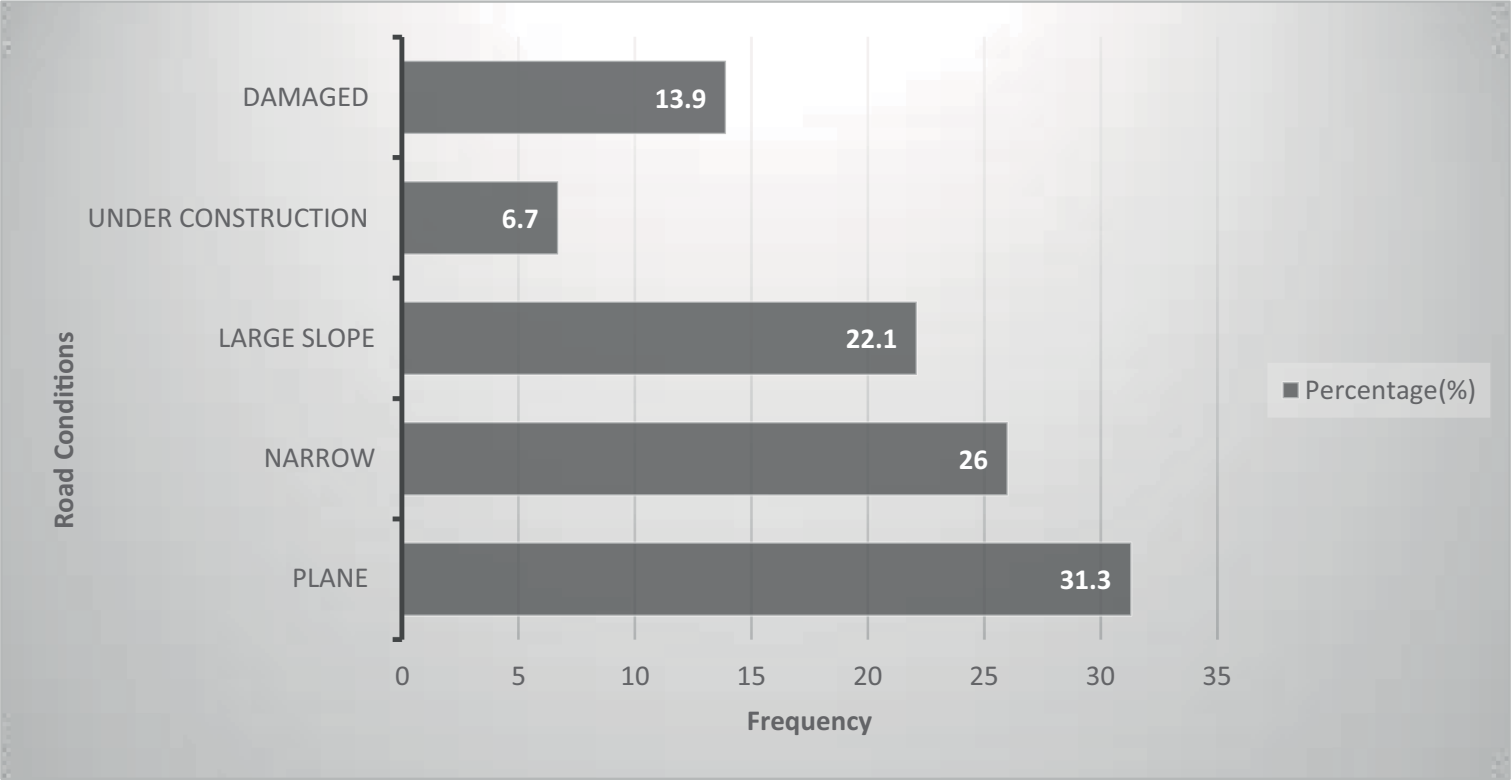
Condition of the roads during an accident among truckers in Gamo zone, southern Ethiopia (*n* = 208).

**Figure 3:**
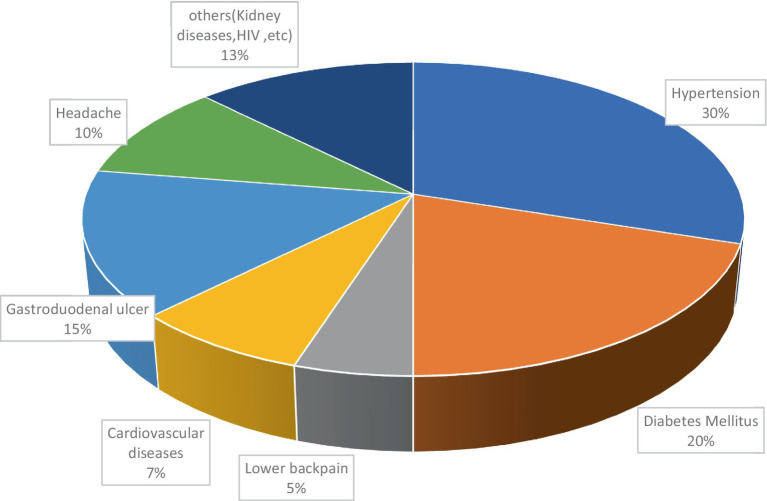
Distribution of diagnosed diseases among truckers in Gamo zone, southern Ethiopia (*n* = 40).

The majority of RTAs occurred on plane roads (31.3%), followed by narrow roads (26%), as shown in Figure 2. Hypertension is relatively high among truckers, followed by diabetes mellitus and gastro-duodenal ulcers at 12 (30%), 8 (20%), and 6 (15%), respectively (Figure 3).

### Near-miss accidents-related factors

**Figure 4:**
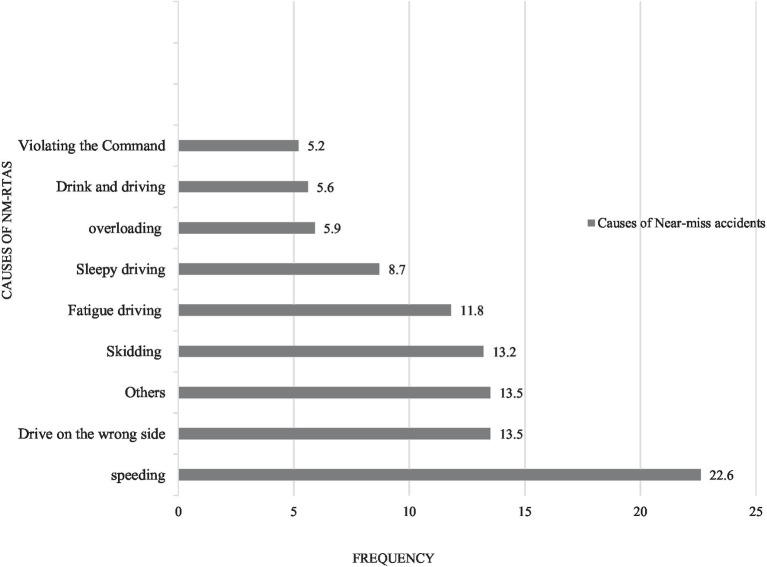
Causes of the near-miss accidents among truckers in Gamo zone, southern Ethiopia, 2022 (*n* = 288).

From the total study participants, more than half 236 (59.4%) of the drivers had a history of driving 8 to 12 h per day, and more than three-fourths of them 316 (79.6%) were driving four and above times per week. Nearly three-fourths 288 (72.5%) of study participants had experienced near-miss accidents. The majority of the near-miss road traffic accidents were caused by speeding, followed by driving on the wrong side of the road and skidding, 65 (22.6%), 39 (13.5%), and 38(13.2%), respectively. Out of the total accidents, about 188 (65.3%) of them happened on the major roads, followed by the street, which accounts for 40 (13.9%). The majority of the accidents 139(48.3%) occurred in foggy weather conditions, followed by rainy weather 87 (30.2%). More than half 237 (59.7%) of the study participants reported that there was no enforcement of the rules and regulations of road safety by traffic police (Table 4; Figure 4).

**Table 4:** Near-miss accident-related factors among truckers in Gamo zone, southern Ethiopia, 2022 (*n* = 397).

Variables	Classifications	Frequency	Percentage (%)
Hours you drive per day	< 8 h	38	9.6
	8–12 h	236	59.4
	≥ 13	123	31.0
How often did you drive per week	Once	5	1.3
	Twice	10	2.5
	Three times	66	16.6
	≥ 4 times	316	79.6
Ever experienced NM-A	Yes	288	72.5
	No	109	27.5
Time of the near-miss accidents	Day time	90	31.3
	Night time	198	68.8
Accident location	Major road	188	65.3
	Street	40	13.9
	Junctions	41	14.2
	Cross road	19	6.6
Condition of the accident road	Damaged	98	34.0
	Narrow	66	22.9
	Large slope	36	12.5
	Wet	21	7.3
	Others (bridge, alternative road,).	67	23.3
Sleeping status, the night before an accident	Not slept the whole night	49	17.0
	Slept for 6 h ≤	162	56.3
	Slept poor quality sleep	77	26.7
Drivers’ health status on accident date	Feeling ill health	47	16.3
	Stressed and drove	67	23.3
	Healthy	174	60.4
Weather condition on the accident date	Sunny	62	21.5
	Rainy	87	30.2
	Foggy	139	48.3
Speed of the truck at accident time	≤ 60 km/h in urban areas	80	27.8
	>60 km/h in urban areas	59	20.4
	≤70 km/h in rural areas	31	10.8
	>70 km/h in rural areas	118	41.0
Lighting condition	Adequate	113	39.2
	Inadequate	175	60.8
Having legal driving license on accident date	Yes	272	94.5
	No	16	5.5
Ever smoked cigarette	Yes	132	33.2
	No	265	66.8
Alcohol taken	Yes	59	20.49
	No	229	79.51
Mobile phone used	Yes	67	23.26
	No	221	76.74
How often do you smoke cigarette?	Daily	92	69.7
	Every other day	6	4.5
	Twice per week	5	3.8
	Used to smoke	24	18.2
	Other (driving time)	5	3.8
History of speeding ticket	Yes	251	63.2
	No	146	36.8
Periodic training opportunities	Yes	314	79.1
	No	83	20.9
Enforcement of the rules and regulations of road safety by police	Yes	160	40.3
	No	237	59.7
Reporting system at your sickness time	Yes	329	82.9
	No	68	17.1
Easy of reporting at time of sickness	Easy	279	70.3
	Difficult	118	29.7

Km/h, Kilometer per hour; NM-A, Near-miss accident.

### Factors associated with near-miss accidents

There were some socio-demographics and near-miss related factors that were significant at 0.25 on binary logistic regression and considered for multivariate logistic regression analysis. These factors included age, education level, marital status, monthly income, hours driven per day, driving frequency per week, location of an accident, road condition, sleeping status, weather condition, having a legal driving license, and ease of the reporting system at the time of the driver’s illness.

After running a multivariate logistic regression analysis, age, driving frequency per week, location of an accident, road condition, sleeping status, and weather conditions were shown to have a significant association with near-miss road accidents at a 5% level of significance.

The current study revealed that the odds of a near-miss accident was 3.769 (95% CI, 2.151, 6.601) times higher among drivers of ≤25 age groups than drivers in greater than 45 age groups. Also, the odds of sustaining a near-miss accident among truck drivers on the major road was 14.574 (95% CI, 4.719, 45.014) times higher as compared to the cross-road location. Likewise, the odds of sustaining a near-miss accident in foggy weather conditions were 5.781 (95% CI, 1.595, 20.961) times higher than in sunny weather conditions (Table 5).

**Table 5:** Factors associated with near-miss accidents among truckers in Gamo zone, southern Ethiopia, 2022 (*n* = 397).

Variables	Classifications	COR (95%CI)	AOR (95%CI)	*p*-Value
Age	≤ 25 years old	0.646(0.313,1.336)	3.769 (2.151, 6.601)	0.000***
	26–45 years old	3.110(1.606, 6.023)	1.376 (0.641, 2.956)	0.413
	> 45 years old	Ref.	Ref	
Educational level	Primary school	2.950(1.507,5.775)	0.834 (0.315, 2.211)	0.715
	Secondary school	1.836(0.878,3.838)	0.963 (0.448, 2.070)	0.923
	College and above	Ref	Ref	
Marital status	Married	Ref	Ref	
	Single	0.609 (0.331,1.119)	1.830 (0.876, 3.821)	0.108
	Divorced	0.320(0.179, 0.571)	1.148 (0.503, 2.620)	0.743
	Widowed	0.502(0.254, 0.989)	0.544 (0.243, 1.217)	0.138
Monthly income	<1,000 ETB	0.417 (0.196, 0.886)	1.102(0.421, 2.882)	0.843
	1,000–3,000 ETB	0.288 (0.144, 0.577)	0.568(0.315, 1.025)	0.061
	3,001–4,000 ETB	0.440 (0.212, 0.915)	0.763 (0.386, 1.508)	0.436
	≥ 4,001 ETB	Ref	Ref	
Hours you drive per day	< 8 h	Ref	Ref	
	8–12 h	1.588(0.821, 3.074)	0.991(0.469, 2.091)	0.980
	≥ 13	2.870(1.356, 6.071)	1.819 (0.791, 4.184)	0.159
How often you drive per week	Once	Ref	Ref	
	Twice	1.418(0.636, 3.158)	1.493(9.641, 3.477)	0.353
	Three times	0.981(0.459, 2.097)	1.272(0.564, 2.870)	0.562
	≥ 4 times	2.835(1.471, 5.465)	3.158(1.561, 6.387)	0.001**
Accident location	Cross road	Ref	Ref	
	Street	2.888 (1.154, 7.226)	1.435(0.655,3.145)	0.367
	Junctions	3.800 (1.492, 9.681)	1.619 (0.784, 3.343)	0.193
	Major road	12.554(5.298,29.747)	14.574(4.719,45.014)	0.000***
Condition of the accident road	Damaged	0.409 (0.184, 0.913)	0.715 (0.370, 1.383)	0.319
	Narrow	0.253 (0.113, 0.568)	0.905 (0.389,2.102)	0.816
	Large slope	0.265 (0.107, 0.656)	0.608 (0.249, 1.485)	0.275
	Wet	0.163 (0.064, 0.420)	4.655 (1.620, 13.381)	0.004**
	Others	Ref	Ref	
Sleeping status, the night before an accident	Not slept the whole night	3.437 (2.044, 5.782)	3.304 (1.081,10.102)	0.036*
	Slept poor quality sleep	0.287 (0.074, 1.111)	3.155 (0.787, 12.653)	0.105
	Slept for 6 h ≤	Ref	Ref	
Weather condition on the accident date	Sunny	Ref	Ref	
	Rainy	2.200 (0.926, 5.224)	1.859 (0.566, 6.114)	0.307
	Foggy	6.950 (2.416, 19.989)	5.781 (1.595,20.961)	0.008**
Having legal driving license	Yes	Ref	Ref	
	No	0.021(0.009, 0.051)	0.030 (0.010, 0.095)	0.000
Easy of reporting system at time of sickness	Easy	Ref		
	Difficult	3.455 (1.421, 8.402)	0.206 (0.036, 1.185)	0.077

AOD, Adjusted odds ratios; CI, Confidence Interval; COR, Crude odds ratio; ETB, Ethiopian Birr; Ref., Reference. Significance Codes: ***0.001, **0.01, *0.05.

## Discussion

The present study aims to assess the near-miss accidents (NM-As) and identify associated factors of accidents among truckers involved in banana transportation in the Gamo zone. The NM-A is the most common road safety-related issue and an important public health problem among drivers. The current study revealed that a high proportion of the truckers involved in banana transportation had sustained NM-As 288 (72.5%). This prevalence of the current study is far higher than the studies conducted in Malaysia 37.5% (39) and Thailand 38.3% (21). The possible reasons for the discrepancies could be the predominance of nighttime working shifts in the study area, longer driving hours during the day, more frequent driving during the week, and differences in road infrastructure between study areas.

In the current study, the majority of accidents (68.8%) occurred at night, which is consistent with the previous study findings done in Malaysia and France (39, 40). The high occurrence of accidents at nighttime could be due to a lack of adequate sleep, fatigued driving, and poor visibility of things while night driving.

Regarding the working hours per day, this study showed that 90.4% of the truckers had working hours greater than or equal to 8 h per day. This is contrary to the study finding from Thailand that about 87.9% of the van drivers had working hours less than 8 h per day (21). The potential difference between the findings would be the variation in the nature of their work. Transporting the passengers might give the drivers an opportunity to take some rest at different transits, at meal times, and while dropping and loading the passengers on the way, could help to lapse sometimes. This could reduce the total working hours per day. But truckers are usually expected to drive longer distances from the initial points to their destinations, usually at night, without having to take breaks, transits, or other activities that will increase total working hours per day. Geographical differences and following diverse road safety rules and regulations could be possible causes of the difference.

This study revealed that the major causes of the NM-As are speeding (22.6%), driving on the wrong side of the road (13.5%), and skidding (13.2%). But the major causes of NM-As in the study done in Japan include heavy traffic (64.1%), poor working conditions like lack of breaks (63%), and lack of road safety education (50%) (22). The disparities between the main causes of accidents may be due to differences in study areas and the socio-demographic characteristics of study participants.

The existence of an association between driver’s age and the occurrence of NMAs has been reported in previous studies (4, 23). Consistent with these studies, being a younger driver was associated with the occurrence of NM-As in this study, AOR 3.769 (95%, CI: 2.151, 6.601). The potential reason for this might be age extremes, either a young driver with less experience or poor performance in attention and global cognition among older drivers.

The likelihood of sustaining the NM-As was approximately three times higher (AOR = 3.158, 95%, CI: 1.561, 6.387) for drivers whose driving frequency was higher per week compared to the lower driving frequency. This finding is consistent with what researchers have reported in previous studies in Japan (20, 23). This could be due to its potential to lead to unsafe driving behaviors.

The scholars reported that the majority of NMAs occur on highways and main roads, at 59.3 and 35.1%, respectively (40). The current study also showed that the vast majority (65.3%) of the NMAs occurred on major roads. Moreover, the probability of sustaining the NMAs on the major road was approximately 15 times higher than at the cross-roads, AOR 14.574 (95%, CI: 4.719–45.014). The possible reasons for the occurrence of accidents on major roads and highways might be due to over speeding and violations of other road safety rules and regulations.

Furthermore, this study found that truckers who were not asleep all night were three times more likely to encounter the NMAs than their counterparts, AOR 3.304 (95%, 1.081, 10.102). This is in line with previous studies (21, 40).

Driving on wet roads and foggy weather conditions were found to be associated with near-miss road traffic accidents. On the other hand, having a chronic disease, not taking enough vacation time, driving long hours per day, continued driving when feeling sick, and difficulty reporting poor health status were found to be significant risks for the NMAs in previous studies (20, 22, 23), but there was no significant association in the current study.

### Limitations of the study

There were some limitations to our study, since we utilized a cross-sectional study design. It must be stressed that a great deal of caution must be exercised in drawing conclusions on causality. Also, the study was done with truckers, which may not be representative of the different types of vehicles drivers use most often during the day. Furthermore, this study attempted to implement the contributory factor interactions model, which has a number of factors that need to be evaluated from various stakeholders. But this study was carried out only among truckers to extract all the necessary data. This may overestimate or underestimate the outcomes, and further study on all factors in the model is recommendable.

## Conclusion

This study reflects that there is a need to address different factors contributing to the NM-As among truckers. The overall prevalence of the NMA was high. Some strongly associated factors were identified. Specifically, the age of the drivers, driving frequency per week, location of an accident, road condition, sleeping status, and weather conditions were significantly associated with near-miss accidents. Subsequently, it is recommended that a road safety program be established, mainly focusing on educating the truckers on risk factors for NMA and its association with actual accidents. Likewise, strengthening existing programs on road safety practices is vital to reducing the occurrence of actual accidents. However, such road safety programs and supervision would be effective if the road safety authorities stressed educating the drivers and undertook close follow-up for the implementation of safe driving behaviors.

Moreover, it would be more recommended if the road safety authorities focused on factors that contribute to the NM-As that are outside the drivers’ related factors. These factors encompass outside factors such as road condition and accident location, organizational influences on the drivers, such as lack of rest and vacation time, and unsafe behaviors such as speeding, overloading, command violations, driving on the wrong side of the road, and drunk-driving.

It is strongly warranted that future researchers could further investigate all contributing factors included in the contributory factors interaction model to identify factors contributing to the NMAs and establish a correlation between the NMAs and actual RTAs.

## Data availability statement

The original contributions presented in the study are included in the article, further inquiries can be directed to the corresponding author.

## Ethics statement

The ethical clearance for the data and the study was approved by the ethical review board of Arba Minch University, with an institutional review board (IRB) number of IRB/1170/2021. After explaining the purpose of the study, an official letter of support from Arba Minch University was brought to the Gamo Zone truck owner’s associations, and permission was obtained. The participant’s informed consent and written consent were obtained from all study participants. Respondents were informed about the purpose of the study, the importance of their participation, and the right to withdraw any time they wished to stop. Privacy and confidentiality issues were also noted. The data was not disclosed to a third party, and it was kept confidential where the investigators had access. The data was totally anonymous and any personal name was not encoded, and the identifiers of the study subjects were simply assigned serial numbers. All the procedures for the data collection were conducted according to the principles of Helsinki.

## Author contributions

TB: Conceptualization, Data curation, Formal analysis, Funding acquisition, Investigation, Methodology, Project administration, Resources, Software, Supervision, Validation, Visualization, Writing – original draft, Writing – review & editing. GT: Conceptualization, Data curation, Resources, Supervision, Writing – review & editing. HL: Conceptualization, Data curation, Formal analysis, Investigation, Methodology, Project administration, Visualization, Writing – review & editing. MM: Conceptualization, Investigation, Methodology, Visualization, Writing – review & editing. MA: Investigation, Methodology, Resources, Software, Writing – review & editing. AO: Conceptualization, Methodology, Writing – review & editing.

## References

[1] World Health Organization. Global status report on road safety 2018: Summary.

[2] World Health Organization. New WHO report highlights insufficient progress to tackle lack of safety on the world’s roads. (2018).

[3] Nations U. United nations conference on trade and development: Transport and Trade facilitation, series no 10. (2017). Available from: http://creativecommons.org/licenses/by/3.0/igo/.

[4] Powell NB Schechtman KB Riley RW Guilleminault C Chiang RPY Weaver EM. Sleepy driver near-misses may predict accident risks. Sleep. (2007) 30:331–42. doi: 10.1093/sleep/30.3.331, PMID: 17425230

[5] Geneva WHO. Save LIVES - a road safety technical package. (2017).

[6] Hayward John C. Near-miss determination through use of a scale of danger. Pennsylvania Transp Traffic Saf Cent. (1972):24–34.

[7] Abegaz T Gebremedhin S. Magnitude of road traffic accident related injuries and fatalities in Ethiopia. (2018). 10.1371/journal.pone.0202240 PMC6350968 30695028

[8] Misker D Tunje A Mengistu A Abera F Yalelet M Gebrie M. Magnitude and factors associated with road traffic accident among traumatized patients in Arba Minch general hospital. Pub Heal Safety. (2017) 2

[9] Zewde T. Determinants that Lead drivers into traffic accidents: a case of Arba Minch City, South Ethiopia. Sci J Appl Math Stat. (2017) 5:210. doi: 10.11648/j.sjams.20170506.13

[10] Chen H Qi H Long R Zhang M. Research on 10-year tendency of China coal mine accidents and the characteristics of human factors. Saf Sci. (2012) 50:745–50. doi: 10.1016/j.ssci.2011.08.040

[11] Dekker SWA. Reconstructing human contributions to accidents: the new view on error and performance. J Saf Res. (2002) 33:371–85. doi: 10.1016/S0022-4375(02)00032-4, PMID: 12404999

[12] World Health Organization. World report on road traffic injury prevention. Geneva, Switzerland. (2004). cited 2023 Jul 4. Available from: https://apps.who.int/iris/bitstream/handle/10665/42925/9241591315.pdf

[13] Amalberti R. The paradoxes of almost totally safe transportation systems. Saf Sci. (2001) 37:109–26. doi: 10.1016/S0925-7535(00)00045-X

[14] Hollnagel E. Barriers and accident prevention. 2nd ed Routledge (2016).

[15] Leveson NG. System safety engineering: Back to the future Massachusetts Institute of Technology (2002).

[16] Filtness A Thomas P Talbot R Quigley C Papadimitriou E Yannis G. The application of systems approach for road safety policy making: Deliverable 8.1 of the H 2020 project safety cube. Loughborough, UK: (2016) [cited 2023 Jul 7]. Available from: https://repository.lboro.ac.uk/articles/report/The_application_of_systems_approach_for_road_safety_policy_making_Deliverable_8_1_of_the_H2020_project_SafetyCube/9353342

[17] Girotto E de Andrade SM González ADMA. Professional experience and traffic accidents/near-miss accidents among truck drivers. Accid Anal Prev. (2016) 95:299–304. doi: 10.1016/j.aap.2016.07.004 27474875

[18] Philip A Corrigan JM Julie WS Erickson M. Near-Miss Analysis In: Patient safety: Achieving a new standard for care. Washington, D.C: National Academies Press (2004). 226–44. 25009854

[19] R Seva R T Flores GM T Gotohio MP C Paras NG. Logit model of motorcycle accidents in the Philippines considering personal and environmental factors. Int J Traffic Transp Eng. (2013) 3:173–84. doi: 10.7708/ijtte.2013.3(2).06

[20] Baba M Miyama G Sugiyama D Hitosugi M. Influence of workplace environment, working conditions and health status of taxi drivers on vehicle collisions or near-miss events. Ind Health. (2019) 57:530–6. doi: 10.2486/indhealth.2018-0104, PMID: 30555104 PMC6685793

[21] Kala W Boonyamalik P Kaewboonchoo O Bandhukul A. Risk factors of near - miss road traffic incidents among inter - provincial public Van drivers in Thailand. J Pub Heal Res Develop. (2019) 10:1396–11. doi: 10.5958/0976-5506.2019.01126.4

[22] Genta M Masakatsu F Ritsuko K Masahito H. Risk factors for collisions and near-miss incidents caused by drowsy bus drivers. Int J Environ Res Public Health. (2020) 17:4370. doi: 10.3390/ijerph17124370 32570777 PMC7345026

[23] Makizako H Shimada H Hotta R Doi T Tsutsumimoto K Nakakubo S. Associations of near-miss traffic incidents with attention and executive function among older Japanese drivers. Gerontology. (2018) 64:495–502. doi: 10.1159/000486547, PMID: 29428957

[24] Halkiyo JB Halkiyu SB Reddy RR. Road accidents of Arba Minch town in Ethiopia: an empirical study. Int J Civ Eng. (2017) 4:29–33. doi: 10.14445/23488352/IJCE-V4I3P107

[25] Zhang Y Liu T Bai Q Shao W Wang Q. New systems-based method to conduct analysis of road traffic accidents. Transp Res Part F Traffic Psychol Behav. (2018) 54:96–109. doi: 10.1016/j.trf.2018.01.019

[26] Sanders RL. Perceived traffic risk for cyclists: the impact of near miss and collision experiences. Accid Anal Prev. (2015) 75:26–34. doi: 10.1016/j.aap.2014.11.004 25460088

[27] Balami AD Sambo G. Road traffic accidents, near-misses and their associated factors among commercial tricycle drivers in a Nigerian city. Health Environ. (2020) 1:1–8.

[28] Toyokuni Y Ishimaru T Honno K Kubo T Matsuda S Fujino Y. Near-miss incidents owing to fatigue and irregular lifestyles in ambulance personnel. Arch Environ Occup Health. (2022) 77:46–50. doi: 10.1080/19338244.2020.1842312, PMID: 33208030

[29] Ajao LA Abisoye BO Jibril IZ Jonah UM Kolo JG. “In-vehicle traffic accident detection and alerting system using distance-time based parameters and radar range algorithm.” In: *2020 IEEE PES/IAS PowerAfrica 2020 Aug 25* (pp. 1–5). IEEE.

[30] Jimoh OD Ajao LA Adeleke OO Kolo SS. A vehicle tracking system using greedy forwarding algorithms for public transportation in urban arterial. IEEE Access. (2020) 8:191706–25. doi: 10.1109/ACCESS.2020.3031488

[31] Shalishe A Bhowmick A Elias K. Meteorological drought monitoring based on satellite CHIRPS product over Gamo zone. Southern Ethiopia Advan Meteorol. (2022) 2022:1–13. doi: 10.1155/2022/9323263

[32] Feyso A Mensa A. Smallholder banana-based farming system dynamics of Arba Minch Zuria District, the case of Gamo zone, Ethiopia: qualitative exploration. Cogent Food Agricul. (2021) 7:1930425. doi: 10.1080/23311932.2021.1930425

[33] Woldu Z Mohammed A Belew D Shumeta Z Bekele A. Assessment of banana production and marketing in Ethiopia. Int J Sci: Basic App Res. (2015) 24:283–307.

[34] Arba-Munch Zuria Distric Agricultural Product Marketing Office. Fruit production and marketing Progress report. Arba-Minch, Ethiopia: (2014).

[35] Rk S Gupta K Kumar A Gk S Verma V Rn S. Developing a precise questionnaire to elucidate risk factors and injury pattern in RTA victims developing a precise questionnaire to elucidate risk factors and injury pattern in RTA victims. (2014).

[36] Bradburn N Rips L Shevell S. Answering autobiographical questions: the impact of memory and inference on surveys. Science, New Series. (1987) 236:157–61. doi: 10.1126/science.3563494 3563494

[37] Grimes D Schulz K. Bias and causal association in observational research. Lancet. (2002) 359:248–52. doi: 10.1016/S0140-6736(02)07451-2 11812579

[38] Jing L Bai Q Guo W Feng Y Liu L Zhang Y. Contributory factors interactions model: a new systems - based accident model. Syst Res Behav Sci. (2019):1–22. doi: 10.1002/sres.2618

[39] Nik Mahdi NNR Bachok N Mohamed N Shafei MN. Risk factors for near miss incident among long distance bus drivers in Malaysia. Iran J Public Health. (2014) 43:117–24.

[40] Sagaspe P Taillard J Bayon V Lagarde E Moore N Boussuge J. Sleepiness, near-misses and driving accidents among a representative population of French drivers. J Sleep Res. (2010) 19:578–84. doi: 10.1111/j.1365-2869.2009.00818.x, PMID: 20408921

